# Age-Related Differences in Trunk Kinematics and Interplanar Decoupling with the Pelvis during Gait in Healthy Older versus Younger Men

**DOI:** 10.3390/jcm12082951

**Published:** 2023-04-18

**Authors:** Alexander Dallaway, Michael Duncan, Corbin Griffen, Jason Tallis, Derek Renshaw, John Hattersley

**Affiliations:** 1 School of Public Health Studies, Faculty of Education, Health and Wellbeing, University of Wolverhampton, Millennium City Building, Wulfruna Street, Wolverhampton WV1 1LY, UK; 2 Coventry NIHR CRF Human Metabolism Research Unit, University Hospitals Coventry and Warwickshire, Clifford Bridge Rd, Coventry CV2 2DX, UK; 3 Centre for Sport, Exercise and Life Sciences, Institute of Health and Wellbeing, Coventry University, Alison Gingell Building, Priory Street, Coventry CV1 5FB, UK; 4 School of Life Sciences, Faculty of Health and Life Sciences, Coventry University, Alison Gingell Building, Priory Street, Coventry CV1 5FB, UK

**Keywords:** 3D motion analysis, walking gait, ageing, back muscles, kinematics

## Abstract

This study investigated age-related differences in trunk kinematics during walking in healthy men. Secondary aims were to investigate the covarying effects of physical activity (PA) and lumbar paravertebral muscle (LPM) morphology on trunk kinematics, and the effect of age on interplanar coupling between the trunk and pelvis. Three-dimensional (3D) trunk and pelvis motion data were obtained for 12 older (67.3 ± 6.0 years) and 12 younger (24.7 ± 3.1 years) healthy men during walking at a self-selected speed along a 10 m walkway. Phase-specific differences were observed in the coronal and transverse planes, with midstance and swing phases highlighted as instances when trunk and pelvic kinematics differed significantly (*p* < 0.05) between the younger group and older group. Controlling for age, fewer significant positive correlations were revealed between trunk and pelvic ranges and planes of motion. LPM morphology and PA were not significant covariates of age-related differences in trunk kinematics. Age-related differences in trunk kinematics were most apparent in the coronal and transverse planes. The results further indicate ageing causes an uncoupling of interplanar upper body movements during gait. These findings provide important information for rehabilitation programmes in older adults designed to improve trunk motion, as well as enable identification of higher-risk movement patterns related to falling.

## 1. Introduction

It is well-known that gait characteristics are modified with ageing, either as a compensatory mechanism to reduce injury risk [1] or as a result of deteriorated muscle activity [2]. Whilst age-induced changes in the biomechanical function of the lower limbs during walking are well understood, the quality and quantity of evidence on age-related adaptations in trunk function are relatively low. This is a likely consequence of clinical gait analyses primarily focusing on lower limb biomechanics [3]. As the proportion of older adults continues to increase globally [4], understanding trunk kinematic changes during gait has become increasingly important and is a critical step in devising effective strategies that preserve independence in older adults.

The trunk muscles, particularly the lumbar paravertebral muscles (LPMs), play a crucial role in walking gait, and actively contribute to dynamic balance during functional activities [5,6]. Dynamic margins of stability are reduced considerably during single-limb support [7]. Therefore, this phase of the gait cycle presents a greater challenge to dynamic stability, particularly in older adults for whom reduced single-limb balance increases the risk of injurious falls [8]. Due to the large mass and moment of inertia of the upper body, particularly the trunk, controlling its position relative to the base of support is critical in maintaining dynamic stability [9]. Older adults may not have the ability to recover from dynamic instability given that their trunk movement control is already being challenged by higher mechanical energy demands, and a forward-leaning position to preserve walking speed and upper body posture [10]. Control of the trunk throughout single limb support is therefore a critical phase in maintaining dynamic stability and reducing falls risk in older adults. Despite this, few studies have considered phase-specific differences in the trunk during the gait cycle as a function of age [10,11,12]. Indeed, previous research [13,14,15] has typically focused on discrete data (e.g., peak trunk flexion angle). To explore the age response in trunk kinematics, analysing phase-specific effects may be more informative than comparing peaks and troughs. Therefore, statistical inferencing methods, such as Statistical Parametric Mapping (SPM), may be more valuable in detecting age-related differences in gait kinematics as the whole time-series waveform is considered.

To separate comorbidities from the effects of ageing, establishing normal age-related changes in trunk kinematics during gait is needed. Therefore, the primary aim of this study was to investigate age-related differences in trunk kinematics during walking between healthy younger and older men. Furthermore, it is important to understand the relationship between degenerative muscle morphology and kinematic changes in the trunk with age, as skeletal muscle atrophy and fat infiltration accompany the ageing process [16,17]. Secondary aims were therefore, to investigate the covarying effects of LPM morphology and physical activity (PA) on trunk kinematics, and the effect of age on interplanar coupling between the trunk and pelvis.

## 2. Materials and Methods

Reporting of this prospective observational case-matched study is based on the Strengthening the Reporting of Observational Studies in Epidemiology (STROBE) Statement [18]. Coventry University Ethics Committee approved the study (P70399) on 13 September 2018.

### 2.1. Participants

Following informed written consent, twenty-four community-dwelling volunteers and university staff and students were stratified into the young group (YG; *n* = 12) or older group (OG; *n* = 12). Inclusion criteria were generally healthy males (e.g., free from disease, musculoskeletal injury or functional impairment) aged between 18 and 30 years, or above 60 years. Exclusion criteria were BMI outside of 18.5–29.9 kg·m^−2^, smokers, consumption of alcohol on a daily basis, use of assistive walking equipment, and an existing or past medical history of metabolic diseases, neuromuscular disorders, or musculoskeletal impairments that may affect muscle function.

To ensure factors such as pain-related disability and PA levels did not confound the results, participants completed the Modified Oswestry Low Back Pain Disability Questionnaire (ODQ-m) [19] and moderate-to-vigorous physical activity (MVPA) was recorded prior to 3D motion analysis using accelerometery. ActiGraph GT9X accelerometers, sampling at 90 Hz, were worn on participants’ dominant wrist for 10 consecutive days based on the recommendations of Migueles et al., [20]. Accelerometer data were processed using ActiLife software (version 6.13). To be eligible for processing, the accelerometer must have been worn for a minimum of 4 days, including one weekend day and at least 10 h of awake time daily [20]. Valid data were divided into 1 s epochs, and a cut-off value of 1031 counts/minute was used to calculate average time spent per day in MVPA [21]. Although no studies have investigated the influence of epoch length on accelerometer outcomes, unpublished data suggest that shorter epochs (1 s vs. 60 s) are more sensitive in detecting time spent in MVPA [20].

### 2.2. 3D Motion Analysis Protocol

Anthropometric measurements and passive retro-reflective marker placement were performed by an experienced researcher (AD). Thirty-nine markers were attached to anatomical landmarks according to the PiG Full Body Marker Model [22] to acquire data on gait cycles and spatiotemporal parameters.

3D motion analysis was performed using a Vicon motion capture system consisting of 14 infrared cameras (Vero2.2, VMS, Oxford, UK) sampling at 100 Hz. All participants walked barefoot along a 10 m walkway at a self-selected speed. Three trials were required to complete data collection for each participant. All gait trials were processed in Vicon Nexus (Version 2.10.1, VMS, Oxford, UK) using standard operations to generate the PiG biomechanical model. Marker trajectory data were low-pass filtered using a 4th order, zero-lag Butterworth filter with a 10 Hz cut-off frequency. The cut-off frequency was based on the recommendations of Sinclair et al. [23], where 99% of the signal power was contained below. Each trial was manually truncated to include one full gait cycle (GC) (heel strike to heel strike) for each limb. Trials were exported into Vicon Polygon (Version 4.4.5, VMS, Oxford, UK) for further analysis. All outcomes were normalised to one GC (100%) using linear interpolation to 101 data samples. Each trial consisted of two GCs, and three trials were processed for each participant (i.e., six steps per participant). Ensemble averages were generated for trunk kinematics in the global reference frame (trunk-G), pelvis kinematics in the global reference frame, and also for trunk kinematics in the pelvic reference frame (trunk-P), as well as spatiotemporal gait parameters.

Mean peak minima and maxima angle data were obtained during the GC for each participant. Mean ROM was also obtained for the trunk-G, trunk-P, and pelvis throughout the GC for each participant. Kinematic data were generated for the trunk and pelvis segments relative to the global reference frame (trunk-G) in the sagittal (anterior/posterior tilt), coronal (obliquity), and transverse (axial rotation) planes. Trunk motion data in the pelvic reference frame (trunk-P), relative rotations between the pelvis and trunk segments were also generated in the sagittal (flexion/extension), coronal (lateral flexion) and transverse (axial rotation) planes. For the trunk-G and pelvis, positive angular values were defined for forward (anterior) bending in tilt, bending to the contralateral side in obliquity, and protraction in axial rotation; negative values represent the opposite movements. Protraction was defined as rotation away from the reference limb, whilst retraction was rotation toward the reference limb. For the trunk-P, positive angular values were defined for flexion in the sagittal plane, bending to the ipsilateral side in the coronal plane, and retraction in the transverse plane; negative values represent the opposite movements.

### 2.3. Muscle Morphology

All participants underwent T2-weighted axial MRI of the lumbar spine. Methods have been described in detail previously [24]. Briefly, normalised muscle volume (NMV) and muscle fat infiltrate (MFI) were determined bilaterally for the LPMs, from the superior endplate L3 to the superior endplate L4, consisting of the psoas, quadratus lumborum, erector spinae, and multifidus. Total LPM volume was normalised to the straight-line distance measured between the anterior superior border of the L1 vertebra and the anterior superior border of the S1 vertebra on T2-weighted mid-sagittal MRI images. MFI was calculated as the mean signal intensity (MSI) of the LPMs as a percentage of the MSI of a homogenous region of subcutaneous back fat.

### 2.4. Statistical Analysis

For spatiotemporal parameters and discrete data (kinematic peaks and ROM), independent sample *t*-tests were performed in SPSS 24.0 (IBM Corp, Armonk, NY, USA) to compare statistical differences between the OG and YG. Muscle morphology (LPM mean MFI and total NMV) and MVPA covariates were assessed using univariate ANCOVA. For each discrete variable, differences between groups were analysed and reported using the ANCOVA test if a significant covariate was found. Zero-order and partial correlations, controlling for age group, were also performed between ROMs in the trunk-G, trunk-P, and pelvis. Alpha level was set at 5% for all statistical tests, and effect sizes (Cohen’s d) calculated where appropriate. All data were normally distributed, as assessed by Shapiro–Wilks test (*p* > 0.05). Where the assumption of homogeneity of variances was violated, as assessed by Levene’s Test of Equality of Variances (*p* < 0.05), the Welch–Satterthwaite correction was used.

To identify phase-specific differences in kinematic waveforms during the GC between age groups, SPM two-tailed independent *t*-tests [25] were performed in MATLAB (R2019a, version 9.6.0.1072779, The MathWorks, Inc., Natick, MA, USA) and implemented using the open-source one-dimensional SPM code (spm1D-package, version 0.4.3, http://spm1d.org/index.html, accessed on 15 July 2020). The primary advantage of SPM is that abstraction of the originally sampled time series does not need to be performed to statistically analyse the data. Interpretation is therefore, more intuitive for time-series data where the statistical result is also a time-series; in this case, a time-series of *t*-values. If instantaneous values of the kinematic waveforms crossed the critical threshold (at which α = 5% of smooth random curves would be expected to traverse), a supra-threshold cluster depicted by grey shading indicated a significant difference (*p* < 0.05) between groups at a specific phase in the GC. Graphical presentation of the kinematic waveforms was performed using GraphPad Prism (Version 8.3.1, San Diego, CA, USA). Data are presented as means with standard deviations (mean ± SD) unless otherwise stated.

## 3. Results

Apart from age, there were no significant differences (*p* > 0.05) in participant characteristics and any of the spatiotemporal gait parameters between the YG and OG (Table 1).

### 3.1. Discrete Measures

There were significant age effects in trunk-G kinematics during the GC, predominantly in the transverse plane. The YG had significantly greater trunk-G ROM than the OG in the sagittal (2.96 ± 0.88° vs. 1.99 ± 0.39°, *p* = 0.002) and transverse planes (6.87 ± 2.21° vs. 5.04 ± 1.29°, *p* = 0.024). Peak trunk-G protraction and retraction were also significantly greater (*p* < 0.05) in the YG compared to the OG (Table 2). All significant differences were large according to effect size estimates (Cohen’s d = 0.9–1.4).

Significant trunk-P kinematic differences between the YG and OG were predominantly in the coronal plane, although transverse plane kinematics also showed large age-related differences despite not reaching significance. Peak trunk-P retraction was significantly greater (*p* = 0.047) in the YG (6.30 ± 1.88°) compared to the OG (4.77 ± 1.65°). The YG also demonstrated greater peak protraction and axial rotation ROM than the OG, although not statistically significant. However, the magnitudes of these differences were large and comparable to that of internal rotation. During stance, the YG (7.19 ± 1.50°) exhibited significantly greater (*p* = 0.004) peak contralateral flexion compared to the OG (5.19 ± 1.64°). The YG (−7.13 ± 1.59°) also demonstrated significantly greater (*p* = 0.005) peak ipsilateral flexion than the OG (−5.03 ± 1.66°) during swing phase, resulting in the YG possessing a lateral flexion ROM 40% greater than the OG (14.31 ± 3.08° vs. 10.22 ± 3.29°, *p* = 0.005). Similar differences were found in pelvic obliquity, where peak upward and downward tilt were significantly greater in the YG compared to the OG (*p* < 0.001). Pelvic obliquity ROM as a result, was also significantly greater (*p* < 0.001) in the YG compared to the OG by 74%. Significant age-related differences in lower back lateral flexion and pelvic obliquity were large according to effect size estimates (Cohen’s d = 1.3–2.0). ANCOVA revealed that MVPA, as well as MFI and NMV of the LPMs, were not significant covariates of any of the discrete spatiotemporal and kinematic variables (*p* > 0.05).

### 3.2. Continuous Measures

**Figure 1:**
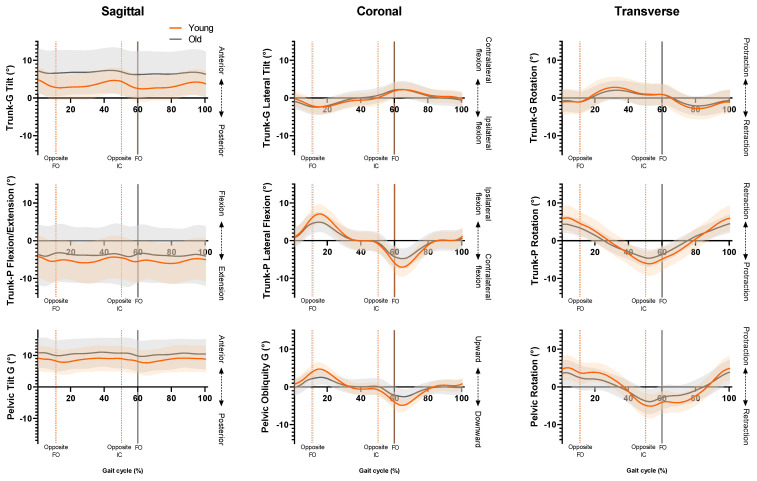
Ensemble averages for trunk-G (trunk relative to global reference frame), trunk-P (trunk relative to pelvic reference frame) and pelvis kinematics. Orange line = YG mean, grey line = OG mean, orange shaded area = YG SD, grey shaded area = OG SD. FO = Foot-off, IC = Initial contact.

**Figure 2:**
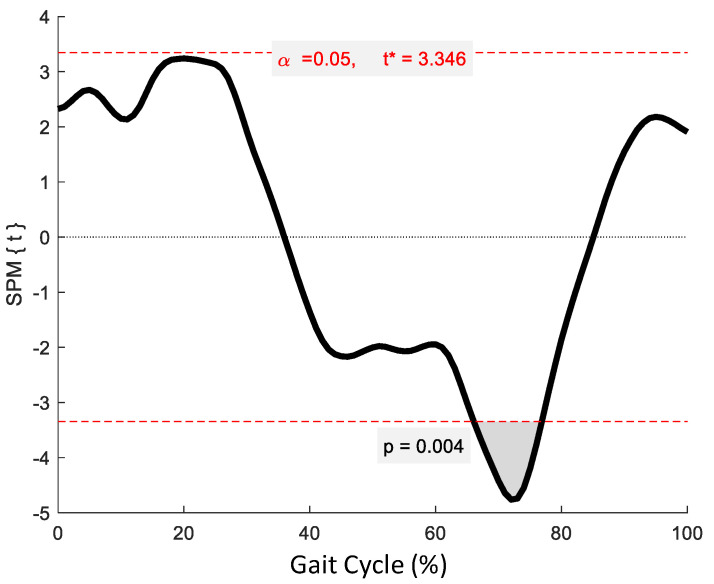
Statistical Parametric Mapping (SPM) output for trunk-P kinematics in the coronal plane. The horizontal red dashed lines represent the critical t* based on α = 0.05 and random field theory calculations of residual smoothness. t* is the critical threshold.

**Figure 3:**
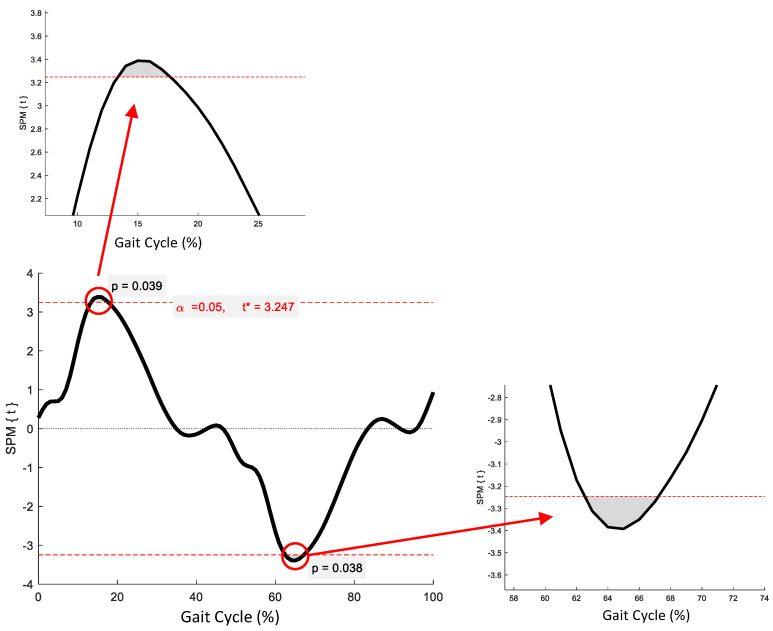
Statistical Parametric Mapping (SPM) output for trunk-P kinematics in the transverse plane. The horizontal red dashed lines represent the critical t* based on α = 0.05 and random field theory calculations of residual smoothness. t* is the critical threshold.

SPM revealed no significant phase-specific differences in trunk-G kinematic waveform patterns between the OG and YG throughout the GC (Figure 1). Two age-related phase-specific differences were identified for trunk-P kinematics in the coronal plane (Figure 2). Two clusters at 13.3–17.7% and 62.5–67.2% exceeded the critical threshold indicating that lateral flexion in the YG was significantly greater during midstance and initial swing than in the OG (*t*(22) = 3.247, *p* = 0.039; *t*(22) = 3.247, *p* = 0.038, respectively). In the transverse plane, one supra-threshold cluster (66.1–76.9%) exceeded the critical threshold of *t*(22) = 3.346 as the YG exhibited significantly greater (*p* = 0.004) trunk-P axial rotation than the OG during swing phase (Figure 3).

**Figure 4:**
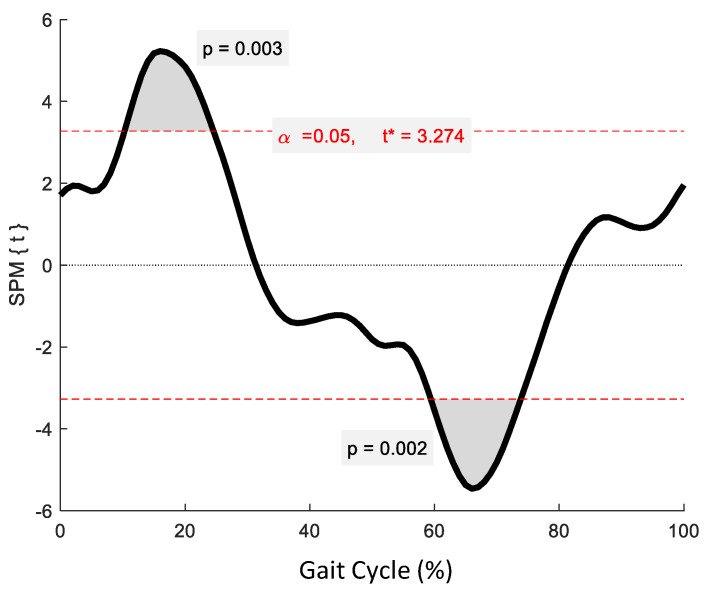
Statistical Parametric Mapping (SPM) output for pelvic kinematics in the coronal plane. The horizontal red dashed lines represent the critical t* based on α = 0.05 and random field theory calculations of residual smoothness. t* is the critical threshold.

**Figure 5:**
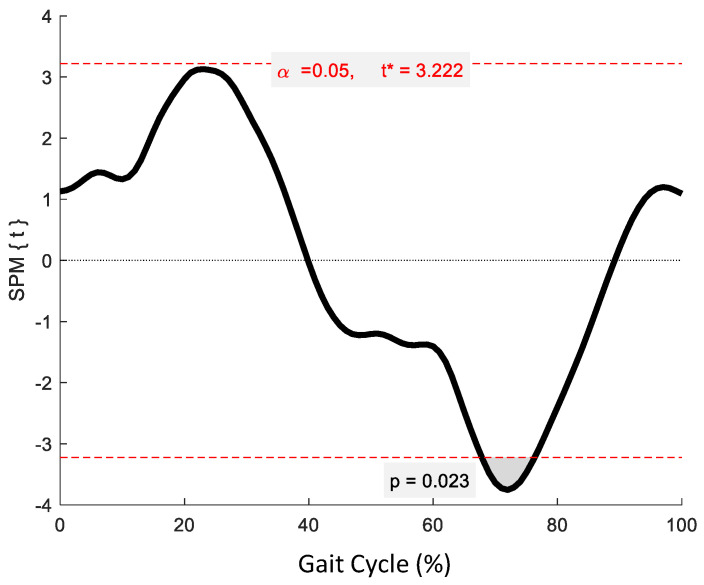
Statistical Parametric Mapping (SPM) output for pelvic kinematics in the transverse plane. The horizontal red dashed lines represent the critical t* based on α = 0.05 and random field theory calculations of residual smoothness. t* is the critical threshold.

No significant phase-specific differences were revealed for pelvic tilt between age groups. Two clusters for pelvic obliquity (Figure 4) at 10.3–24.6% and 59.4–73.8% exceeded the critical threshold indicating that pelvic obliquity in the YG was significantly greater during midstance and initial swing than in the OG (*t*(22) = 3.274, *p* = 0.003; *t*(22) = 3.247, *p* = 0.002, respectively). One supra-threshold cluster (67.8–76.3%) exceeded the critical threshold of *t*(22) = 3.222 for pelvic axial rotation, as the YG exhibited significantly greater pelvic rotation than the OG during swing phase (*p* = 0.023) (Figure 5).

### 3.3. Correlations between Planes of Motion and Intersegment Motion

There were multiple significant interplanar and intersegment correlations in ROMs (Table 3). However, after controlling for age group, only significant partial correlations remained between trunk-P lateral flexion ROM and trunk-G lateral tilt ROM (r(21) = 0.43, *p* = 0.043), trunk-P lateral flexion ROM and pelvic obliquity ROM (r(21) = 0.56, *p* = 0.005), and trunk-P axial rotation ROM and pelvic axial rotation ROM (r(21) = 0.63, *p* = 0.001).

## 4. Discussion

This study identifies age-related differences in trunk kinematics between healthy older men and younger counterparts. To date, differences in trunk kinematics between younger and older participants have not been fully examined. As a consequence, the present study adds new knowledge in relation to ageing gait biomechanics to the literature base. The key findings from the present study were that age-related differences in trunk-P kinematics were greatest in the coronal and transverse planes, and that the initial periods of single limb support were identified as important phases during the GC, where significant differences in trunk kinematics between younger and older adults were detected. This suggests that age-related differences in the trunk are most apparent when dynamic balance is compromised during initial single limb support phases. Given that trunk kinematics are important to locomotor control and maintaining balance in older age [26], these observations highlight the importance of analysing the upper body during gait, and have potential benefits with regards to identifying falls risk in older adults.

### 4.1. Age-Related Changes in the Sagittal Plane

Sagittal plane trunk-G and pelvis kinematics were similar between the OG and YG, resulting in similar trunk-P movement patterns. However, there was a delayed phase shift in the YG, resulting in altered phase-specific flexion/extension movements of the trunk-P. Trunk-G tilt exhibited a biphasic oscillation, corresponding to one flexion/extension cycle for each step. These findings are supported by other gait studies [27,28], and substantiated by EMG studies that have shown peaks of paraspinal muscle activity at early midstance and around foot-off [29,30]. Indeed, ES muscle activity precedes corresponding kinematics indicating that the paravertebral muscles drive trunk movement by anticipating propulsive phases in walking [5].

The OG adopted a more forward-tilted trunk-G throughout the GC, possibly to account for reduced propulsive force [31]. A similar strategy has been observed in lower-extremity amputees [32]. According to Leroux et al., [33] tilting the trunk forward is the best strategy to generate greater forward propulsion during walking gait. Therefore, the OG may have adopted a greater anterior trunk-G angle, causing an anterior shift in centre of mass (COM) to facilitate their forward progression. Tilting the trunk anteriorly may also play an injury prevention role, reducing lower limb stress by damping COM oscillations [26]. The OG also exhibited significantly less trunk-G ROM than the YG. Reduced trunk-G ROM may be indicative of a conservative gait strategy to prevent larger destabilising forces in the sagittal plane [11], despite this being less efficient as more muscular work would be required for horizontal displacement of the COM.

### 4.2. Coronal Plane Changes in Older Age

Trunk motion in the coronal plane was less variable than in the sagittal plane and similar between age groups. Results from the SPM indicate that there is a phase-specific age effect in trunk-P coronal plane kinematics during the early midstance and early swing phase. This is supported by movement amplitudes being significantly greater in the YG than the OG. Given that trunk-G movements were similar in phase and amplitude between groups, and pelvic obliquity exhibited similar significant differences to trunk-P lateral flexion, it is likely that trunk-P motion in the coronal plane was simply a reflection of pelvic movement. This is supported by Crosbie, et al., [28] who state that spinal movements associated with walking are linked to the primary motions of the pelvis.

Pelvic motion in the coronal plane is particularly important for reducing trunk-G oscillations, which could excessively displace COM and cause lateral instability during walking. Instability during the GC may be minimised by permitting a relatively larger ROM in the trunk relative to the pelvis than in the global reference frame through the independent motions of the pelvis. The reduced pelvic, and thus trunk-P ROM in the OG may decrease stability during gait, and indeed explain why falls are more prevalent in the elderly [34]. Lower lateral flexion peaks in the OG may also lead to reduced foot clearance [35] and consequently increase the risk of falling [36]. However, older adults may prevent impact injuries by decreasing trunk movement in the coronal plane. Reducing peak lateral flexion during the early stages of single-limb support allowed the OG to decrease trunk-P angular velocity when it started to move towards the opposite side, which may be an effort to reduce impact from heel strike [14].

### 4.3. Kinematic Changes in the Transverse Plane

Whilst it appears that trunk-G axial rotation was reduced by ageing based on peak values and ROM, no phase-specific differences were observed. Furthermore, coinciding phase-specific differences between groups were identified in the pelvis (68–76% of GC) and trunk-P (66–77% of GC) during swing phase. Coupling between the trunk-P and pelvis was further supported by the significant correlation in their ROMs in the transverse plane. These results indicate that age-related changes in the transverse plane involve a complex interrelationship between the trunk and pelvis. It appears that instances of peak rotational excursion during gait immediately following heel strikes are affected by age-related kinematic changes in the trunk-G. Whereas, during single limb support, age-related differences in the pelvis appear to be more influential than in the trunk-G to trunk-P motion.

### 4.4. Age Effect on Interplanar Motions

The results of the present study suggest there is a change in planar motion with ageing. Zero-order correlations showed that there were numerous significant relationships between the ROM in the trunk-P, trunk-G, and pelvis in different planes. After controlling for age, only three significant correlations remained (trunk-P lateral flexion with trunk-G lateral tilt, trunk-P lateral flexion with pelvic obliquity, and trunk-P axial rotation with pelvic axial rotation), all exclusive to their respective planes. This indicates that ageing causes rotations of the upper body in any given plane to become independent of rotations in orthogonal planes. This finding is novel, although others have shown that coronal and transverse plane trunk motions are interconnected in younger adults [14,37], and that older adults exhibit reduced compensatory coordination between trunk and pelvis movements during walking [11].

In older age, disassociation of trunk and pelvic movements may increase the energetic demands of walking if angular momentum cannot be conserved from motions in orthogonal planes to assist in the forward progression of the body’s COM [38,39]. Whilst the exact mechanisms for the age-related disassociation of interplanar motions in the trunk are unknown, it has been suggested that the vector of the spinal muscles may be responsible for the association between coronal and transverse plane trunk movement [14]. Therefore, including data on muscle fibre orientation derived by MRI diffusion-tensor techniques [40] may elucidate mechanisms. Macroscopic features such as MFI and NMV are not likely responsible for interplanar decoupling in older age based on the current results.

### 4.5. Clinical and Practical Applications

In clinical gait analysis, the upper body is typically overlooked whilst the lower limbs receive much of the focus. Data from the present study indicate that the upper body’s contribution should be considered during gait to inform the design of more effective strategies to preserve mobility in older age. Understanding age-related changes in trunk biomechanics could assist clinical decision making and public health strategies with regards to incorporating motor skills training into exercise interventions and PA programmes. Exercise interventions generally focus on promoting strength, endurance, balance, and flexibility in older adults. Whilst useful, incorporating trunk motor skills training may improve physical function and mobility to a greater extent. The current findings could support a targeted approach by identifying movement patterns in the trunk which should be prioritised to improve physical function in older age. Routinely analysing the trunk will also increase the evidence base and establish normative data, which could be used to identify abnormal movement patterns that may not be apparent in the lower limbs. Furthermore, the full-body PiG model would be appropriate in clinical settings where a balance of accuracy and practicality is needed.

### 4.6. Limitations

There are limitations in this study that should be acknowledged. Whilst PiG has been used to estimate lumbar spine kinematics as the intersection between the modelled trunk and pelvic segments [14,41], it provides a gross understanding of trunk kinematics. More complex spinal models may offer greater accuracy at individual vertebral levels. However, they may also increase measurement error as the size of functional spinal units is small and limits our ability to position three non-colinear markers on the skin overlying multiple functional spinal units [42]. The increased data collection and computational requirements may also make more detailed approaches less clinically applicable [43]. Confidence in the current results is high since the movement patterns and peak values in all planes were highly comparable to a study using indwelling bone pins to assess the 3D motion of the lumbar spine during gait [44], which is considered the gold-standard approach. It should be noted that the current modelling approach for the pelvis, whilst commonly used, may introduce systematic error and a false anterior tilt. Normalising anterior pelvic tilt to zero by defining a pelvis angle that has a coronal plane parallel to the floor may be more convenient for describing pelvic motion.

Secondly, the cohort recruited in the current study comprised healthy, active, young, and older adult men. It has been shown that trunk kinematics are affected by disease and physical impairment such as stroke [41]. Therefore, caution should be taken when generalising the findings of the current study to populations other than healthy men. This study was also specific to walking gait biomechanics on a level surface. Age-related differences may be more pronounced for more challenging movements such as stair negotiation and sit-to-stand.

Group sample sizes were also somewhat smaller than the number generally targeted in most gait studies. This study was part of a larger project in which the MRI parameters were the primary outcome measures. Therefore, the a priori power calculation was based on MRI outcomes, which indicated a minimum of *n* = 8 participants per group was sufficient based on moderate to large effect sizes (α error = 0.05, β error = 0.8). Regarding the implications of sample size for SPM in the current study, it is likely that the analysis was underpowered to detect subtle differences in kinematics between groups. Research has shown that required sample sizes range widely from five to over thirty participants per group to detect differences of 10° to 5° using SPM methods [45]. Detecting more subtle differences (e.g., 2°) between groups would require an even greater sample size to ensure sufficient statistical power, which is atypical in biomechanical research.

## 5. Conclusions

This study offers new insight by providing trunk motion data in all cardinal planes and using one-dimensional SPM to uncover phase-specific differences with respect to ageing. In this study, ageing modified trunk kinematics primarily in the coronal and transverse planes, although this may be a reflection of changes in pelvic motion. Age-related kinematic changes were characterized by reductions in peak amplitudes and ROM in all planes, which were likely to be manifestations of a conservative gait strategy. Muscle degeneration in the lumbar spine and MVPA did not covary with age-related changes in trunk kinematics. Future research should focus on a range of age groups and diseased populations to further understand normal trunk motion during gait with ageing.

## Figures and Tables

**Table 1:** Participant characteristics and spatiotemporal parameters during normal gait (mean ± SD) for the younger and older groups.

Parameter	Younger Group (*n* = 12)	Older Group (*n* = 12)	Independent *t*-Test	Cohen’s d
Participant characteristics				
Age (years)	24.7 ± 3.1	67.3 ± 6.0	*t*(22) = −21.8, *p* < 0.001	8.92
Height (m)	1.78 ± 0.1	1.74 ± 0.1	*t*(22) = 1.2, *p* = 0.23	0.40
Mass (kg)	76.4 ± 11.2	79.2 ± 10.8	*t*(22) = −0.6, *p* = 0.55	0.25
BMI (kg·m^−2^)	24.1 ± 2.2	26.0 ± 2.7	*t*(22) = −1.9, *p* = 0.07	0.77
ODQ-m (%)	2.2 ± 2.3	2.2 ± 3.5	*t*(22) = 0.0, *p* = 1.00	0.00
MVPA (hours per day)	6.6 ± 1.4	6.3 ± 1.5	*t*(22) = 0.5, *p* = 0.60	0.21
Spatiotemporal data				
Walking Speed (m·s^−1^)	1.45 ± 0.19	1.33 ± 0.16	*t*(22) = 1.70, *p* = 0.10	0.69
Cadence (steps·min^−1^)	113.9 ± 5.8	113.0 ± 7.2	*t*(22) = 0.34, *p* = 0.74	0.14
Step Length (m)	0.76 ± 0.08	0.71 ± 0.06	*t*(22) = 1.85, *p* = 0.08	0.76
Normalised Step Length	0.43 ± 0.05	0.41 ± 0.03	*t*(19.7) = 1.43, *p* = 0.17	0.59
Step Width (m)	0.14 ± 0.03	0.16 ± 0.03	*t*(22) = −1.22, *p* = 0.23	0.50

Note: italics denote significant age effect, MVPA = moderate-to-vigorous physical activity, ODQ-m = Modified Oswestry Low Back Pain Disability Questionnaire, Normalized Step Length was relative to height in meters.

**Table 2:** Trunk and pelvic kinematic peaks and ROMs (mean ± SD) for the younger and older groups during normal walking gait.

Parameter	Younger Group (*n* = 12)	Older Group (*n* = 12)	Independent *t*-Test	Cohen’s d
Trunk-G (°)				
Antero-posterior Tilt ROM **	2.96 ± 0.88	1.99 ± 0.39	*t*(22) = 3.49, *p* = 0.002	1.43
Max Anterior Tilt	5.08 ± 3.21	7.69 ± 6.10	*t*(22) = −1.31, *p* = 0.20	0.54
Min Anterior Tilt	2.12 ± 3.34	5.70 ± 6.29	*t*(22) = −1.74, *p* = 0.10	0.71
Lateral Tilt ROM	4.78 ± 2.28	4.95 ± 2.82	*t*(22) = −0.16, *p* = 0.88	0.06
Contralateral Flexion	2.30 ± 1.18	2.38 ± 1.44	*t*(22) = −0.15, *p* = 0.88	0.06
Ipsilateral Flexion	−2.48 ± 1.11	−2.56 ± 1.39	*t*(22) = 0.16, *p* = 0.87	0.07
Axial Rotation ROM *	6.87 ± 2.21	5.04 ± 1.29	*t*(17.7) = 2.48, *p* = 0.02	1.01
Protraction Rotation *	3.42 ± 1.06	2.50 ± 0.58	*t*(16.9) = 2.64, *p* = 0.02	1.08
Retraction Rotation *	−3.45 ± 1.19	−2.54 ± 0.74	*t*(22) = −2.25, *p* = 0.04	0.92
Trunk-P (°)				
Flexion/Extension ROM	2.85 ± 1.00	2.23 ± 0.69	*t*(22) = 1.76, *p* = 0.09	0.72
Max Extension	−6.45 ± 5.32	−4.87 ± 7.79	*t*(22) = −0.58, *p* = 0.57	0.24
Min Extension	−3.61 ± 5.31	−2.64 ± 7.82	*t*(22) = −0.35, *p* = 0.73	0.14
Lateral Flexion ROM **	14.31 ± 3.08	10.22 ± 3.29	*t*(22) = 3.15, *p* = 0.005	1.29
Ipsilateral Flexion **	7.19 ± 1.50	5.19 ± 1.64	*t*(22) = 3.10, *p* = 0.005	1.27
Contralateral Flexion **	−7.13 ± 1.59	−5.03 ± 1.66	*t*(22) = −3.18, *p* = 0.004	1.30
Axial Rotation ROM	12.51 ± 3.85	9.55 ± 3.20	*t*(22) = 2.05, *p* = 0.05	0.84
Protraction Rotation	−6.21 ± 1.99	−4.78 ± 1.56	*t*(22) =−1.97, *p* = 0.06	0.80
Retraction Rotation *	6.30 ± 1.88	4.77 ± 1.65	*t*(22) = 2.11, *p* = 0.05	0.86
Pelvis (°)				
Antero-posterior Tilt ROM	2.31 ± 0.75	2.28 ± 0.65	*t*(22) = 0.11, *p* = 0.91	0.05
Max Anterior Tilt	9.72 ± 4.00	11.60 ± 4.96	*t*(22) = −1.02, *p* = 0.32	0.42
Min Anterior Tilt	7.41 ± 4.04	9.32 ± 4.97	*t*(22) = −1.03, *p* = 0.31	0.42
Obliquity ROM ***	9.83 ± 2.45	5.64 ± 1.72	*t*(22) = 4.85, *p* < 0.001	1.98
Upward Tilt ***	4.87 ± 1.23	2.84 ± 0.81	*t*(22) = 4.77, *p* < 0.001	1.95
Downward Tilt ***	−4.96 ± 1.23	−2.80 ± 0.94	*t*(22) = −4.83, *p* < 0.001	1.97
Axial Rotation ROM	11.92 ± 4.35	8.62 ± 3.82	*t*(22) = 1.98, *p* = 0.06	0.81
Protraction Rotation	6.01 ± 2.25	4.39 ± 1.93	*t*(22) = 1.90, *p* = 0.07	0.77
Retraction Rotation	−5.91 ± 2.12	−4.23 ± 1.93	*t*(22) = −2.03, *p* = 0.05	0.83

Age effect significance values * *p* < 0.05, ** *p* < 0.01, *** *p* < 0.001; ROM = range of motion.

**Table 3:** Zero-order correlation coefficients for ROM between the trunk-P, trunk-G, and pelvis in all cardinal planes.

		Trunk-P ROM			Trunk-G ROM			Pelvic ROM		
		Flexion/ Extension	Lateral Flexion	Axial Rotation	Tilt	Lateral Tilt	Axial Rotation	Tilt	Obliquity	Axial Rotation
Trunk-P ROM	Flexion/Extension	r = 1.0	r = 0.43 *	r = 0.37	r = 0.03	r = 0.14	r = 0.41 *	r = 0.23	r = 0.37	r = 0.27
	Lateral flexion		r = 1.0	r = 0.25	r = 0.46 *	r = 0.33	r = 0.44 *	r = 0.29	r = 0.73 **	r = 0.06
	Axial rotation			r = 1.0	r = 0.12	r = 0.14	r = 0.45 *	r = 0.02	r = 0.37	r = 0.69 **
Trunk-G ROM	Tilt				r = 1.0	r = −0.02	r = 0.25	r = 0.13	r = 0.46 *	r = 0.14
	Lateral tilt					r = 1.0	r = 0.31	r = 0.01	r = −0.13	r = −0.14
	Axial rotation						r = 1.0	r = −0.30	r = 0.39	r = 0.44 *
Pelvic ROM	Tilt							r = 1.0	r = 0.22	r = −0.02
	Obliquity								r = 1.0	r = 0.43 *
	Axial rotation									r = 1.0

* *p* < 0.05, ** *p* < 0.001.

## Data Availability

The data presented in this study are not publicly available due to privacy or ethical restrictions.
